# Molecular Basis of Plant Oil Biosynthesis: Insights Gained From Studying the WRINKLED1 Transcription Factor

**DOI:** 10.3389/fpls.2020.00024

**Published:** 2020-02-04

**Authors:** Que Kong, Yuzhou Yang, Liang Guo, Ling Yuan, Wei Ma

**Affiliations:** ^1^School of Biological Sciences, Nanyang Technological University, Singapore, Singapore; ^2^National Key Laboratory of Crop Genetic Improvement, Huazhong Agricultural University, Wuhan, China; ^3^Department of Plant and Soil Sciences, Kentucky Tobacco Research and Development Center, University of Kentucky, Lexington, KY, United States

**Keywords:** WRI1, transcription factor, plant oil biosynthesis, intrinsically disordered region, post-translational modifications, protein stability, protein–protein interaction

## Abstract

Most plant species generate and store triacylglycerol (TAG) in their seeds, serving as a core supply of carbon and energy to support seedling development. Plant seed oils have a wide variety of applications, from being essential for human diets to serving as industrial renewable feedstock. WRINKLED1 (WRI1) transcription factor plays a central role in the transcriptional regulation of plant fatty acid biosynthesis. Since the discovery of *Arabidopsis WRI1* gene (*AtWRI1*) in 2004, the function of WRI1 in plant oil biosynthesis has been studied intensively. In recent years, the identification of WRI1 co-regulators and deeper investigations of the structural features and molecular functions of WRI1 have advanced our understanding of the mechanism of the transcriptional regulation of plant oil biosynthesis. These advances also help pave the way for novel approaches that will better utilize WRI1 for bioengineering oil production in crops.

## Introduction

Plants biosynthesize and store fatty acids mostly as triacylglycerol (TAG) in their seeds to support seedling development as carbon and energy resource. TAG (often familiar to many people as vegetable oils) is a highly energy-rich natural resource, as it has higher energy compared to carbohydrates and proteins. Vegetable oils are not only vital for the human diet, but also have other important applications such as the production of detergents and lubricants. Vegetable oils are also used to produce biodiesel. The global demand for plant oils is rapidly increasing, and is estimated to double by 2030 (Chapman and Ohlrogge, 2012). The growing demand for vegetable oil increases the need for higher plant oil production.

TAG biosynthesis consists of two major steps, fatty acid biosynthesis and TAG assembly, requiring the collaboration between cellular compartment plastids and the endoplasmic reticulum (ER) (Ohlrogge and Chapman, 2011). Fatty acids are first synthesized in the plastids and then exported to ER to complete the TAG assembly. Fatty acid biosynthesis initiates with acetyl-CoA carboxylase (ACC) that catalyzes the carboxylation to convert acetyl-CoA and bicarbonate into malonyl-CoA. Fatty acids are next elongated by the fatty acid synthase (FAS) complex, with two carbon increments. Upon completion of fatty acids assembly, mediated by FAS, the synthesized fatty acids are exported from plastids to ER, in forms of acyl-CoA. The subsequent TAG synthesis in ER mainly occurs through the eukaryotic phospholipid biosynthetic pathway (Bates et al., 2009). In the last step of TAG synthesis, diacylglycerol (DAG) is converted to TAG (either using acyl-CoA or phospholipids), which is catalyzed by diacylglycerol acyltransferase (DGAT) or phosphatidylcholine:diacylglycerol acyltransferase (PDAT) (Zou et al., 1999; Dahlqvist et al., 2000; Zhang et al., 2009). TAG biosynthesis is sophisticated and not fully understood; therefore, investigation of regulatory mechanisms of TAG biosynthesis is essential, not only for advancing the basic research of plant lipids, but also for bioengineering novel oil crops with increased oil content.

## Crucial Role of WRI1 in Transcriptional Control of Plant Oil Biosynthesis

**Figure 1:**
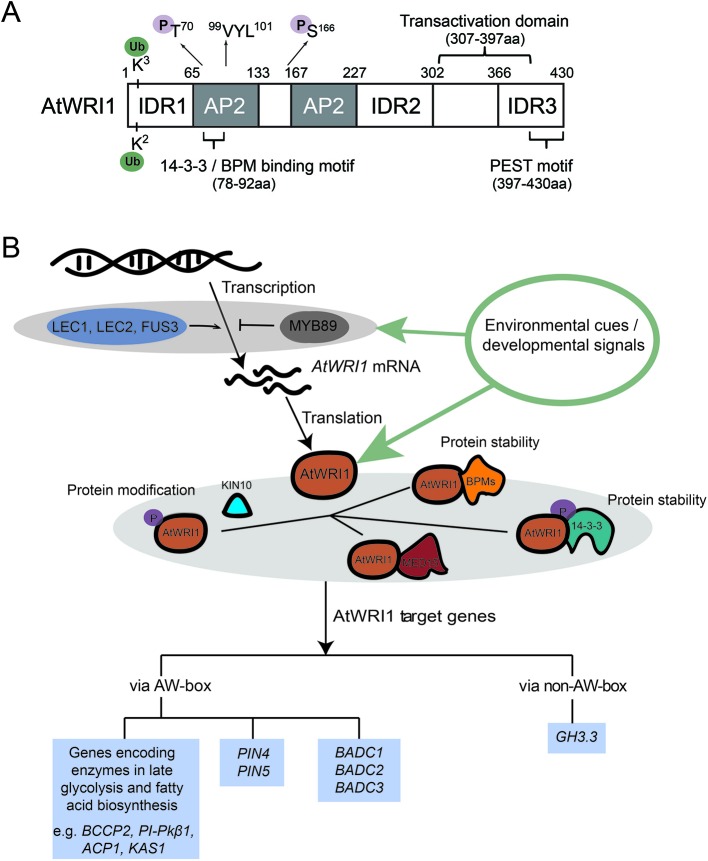
AtWRI1 structural characteristics and molecular mechanism of AtWRI1-regulated gene expression. **(A)** Schematic diagram of AtWRI1. AtWRI1 protein comprises of two AP2 domains, three intrinsically disordered regions (IDRs), a functional motif of “VYL”, the transactivation domain (TAD), the 14-3-3 and E3 ligase adaptor (BPM) binding motifs, the PEST motif, the ubiquitination sites, and the KIN10 phosphorylation sites. **(B)** Regulatory mechanism of AtWRI1 target genes. LEC1, LEC2, and FUS3 are positive regulators which mediate *AtWRI1* expression. MYB89 acts as a negative regulator of *AtWRI1* expression. At the protein level, AtWRI1 is regulated by post-translational modifications (such as phosphorylation and ubiquitination) and AtWRI1-interacting regulators, such as 14-3-3s, mediator subunit 15 (MED15), BPMs, and kinase KIN10. In the perception of environmental or developmental signals, the regulators fine-tune the protein stability and transcriptional activity of AtWRI1, through protein complex assembly and modifications, leading to mediation of the expression of AtWRI1 target genes.

Numerous studies have shown that WRINKLED1 (WRI1) is vital for transcriptional control of plant oil biosynthesis. The discovery of *wri1-1* mutant (the *Arabidopsis* loss-of-function mutant of *AtWRI1*) was reported in 1998, leading to subsequent characterization of AtWRI1. Compared to the wild-type (WT), seed oil content in *wri1-1* is reduced by 80% (Focks and Benning, 1998). The *WRI1* gene encodes a transcription factor which belongs to the APETALA2 (AP2) transcription factor family (**Figure 1A**) (Cernac and Benning, 2004; Masaki et al., 2005). Transcriptomic (microarray) analysis of WT and *wri1-1* using developing seeds found that the majority of genes with reduced expression level in the *wri1-1* mutant encode enzymes in late glycolysis and fatty acid biosynthesis (Ruuska et al., 2002). Subsequent studies confirmed that a number of genes in late glycolysis and fatty acid biosynthesis are indeed AtWRI1 targets (Baud et al., 2007; Maeo et al., 2009). The AW-box ([CnTnG](n)_7_[CG]) was characterized as an AtWRI1 binding element (Maeo et al., 2009). WRI1 was hence considered a “master regulator” in transcriptional control of TAG biosynthesis (Chapman and Ohlrogge, 2012).

## Regulators Controlling the Expression of *WRI1*

Two central seed developmental regulators, LEAFY COTYLEDON1 (LEC1) and LEAFY COTYLEDON2 (LEC2), were hypothesized as essential transcriptional regulators which regulate *AtWRI1* expression (**Figure 1B**). Expression of *AtWRI1* is increased in the *LEC1* gain-of-function mutant, *tnp* (Casson and Lindsey, 2006). In addition, expression of *AtWRI1* is elevated in transgenic plants overexpressing *LEC1* (Mu et al., 2008), suggesting LEC1 is possibly an upstream transcriptional regulator which activates the expression of *AtWRI1* (Santos-Mendoza et al., 2008; Marchive et al., 2014). Induction of *LEC2* in inducible *LEC2*-overexpression lines leads to the activation of *AtWRI1*, whereas *AtWRI1* expression is decreased in *lec2* mutant compared to WT plants (Baud et al., 2007). Therefore, the genetic and molecular evidence indicates that LEC2 functions as another transcriptional regulator controlling *AtWRI1*. In addition, transgenic maize overexpressing maize *LEC1* (*ZmLEC1*) results in increased expression of maize *WRI1* (*ZmWRI1*). ZmLEC1 is also shown to increase the *GUS* expression of *_promoter_ZmWRI1:GUS* in maize cell culture (Shen et al., 2010). Transgenic soybean plants overexpressing soybean *LEC2* (*GmLEC2*) results in elevated expression of soybean *WRI1* (*GmWRI1*) (Manan et al., 2017). The evidence suggested a conserved mechanism controlling *WRI1* expression *via* LEC1 and LEC2 in different plant species. How LEC1 or LEC2 binds the AtWRI1 promoter has not been determined (Santos-Mendoza et al., 2008; Marchive et al., 2014). Importantly, recent ChIP followed by DNA microarray (ChIP-chip) experiments demonstrated that *AtWRI1* is a direct target gene of LEC1 (Pelletier et al., 2017).

FUSCA3 (FUS3) is another transcriptional regulator which mediates the expression of *AtWRI1* (**Figure 1B**). Comparative transcriptome analysis using microarray showed that expression of *AtWRI1* is reduced in a *fus3* mutant (Yamamoto et al., 2010). FUS3 has been speculated to regulate the expression of *AtWRI1* similar to LEC2, creating the functional redundancy between FUS3 and LEC2 (Yamamoto et al., 2010). Recent ChIP-Chip evidence confirmed that *AtWRI1* is a direct target of FUS3 (Wang and Perry, 2013). Sucrose treatment induced *AtWRI1* expression (Masaki et al., 2005). Expression of some sugar-responsive genes is stimulated by AtWRI1, suggesting the potential role of WRI1 in sugar signaling from carbon flow to oil production (Masaki et al., 2005). FUS3 does not affect *LEC1/LEC2* expression, suggesting that activation of *AtWRI1* by sucrose is *via* FUS3 instead of LEC1/LEC2 (Zhang et al., 2016a).

In addition to the transcriptional regulators LEC1, LEC2, and FUS3, other plants species may have alternative upstream regulators which contribute to mediating the expression of *WRI1*. In oil palm mesocarp, where most palm oil is derived from and *EgWRI1* displays high expression, the inability to detect expression of key regulator genes, such as *EgLEC1*, *EgLEC2*, and *EgFUS3*, suggests that expression of *EgWRI1* involves additional fruit-specific upstream regulators (Bourgis et al., 2011). EgNF-YA3, EgNF-YC2, and EgABI5 have been recently found to be capable of binding to the promoter region and activate the expression of *EgWRI1* (Yeap et al., 2017).

Arabidopsis transcription factor MYB89 is a newly identified transcriptional repressor of *AtWRI1* (**Figure 1B**). Transgenic plants overexpressing *MYB89* shows decreased *AtWRI1* expression, and a *myb89* loss-of-function mutant displays elevated *AtWRI1* expression compared to WT (Li et al., 2017a). ChIP experiments also showed that MYB89 binds to the *AtWRI1* promoter directly, suggesting that *AtWRI1* is a direct target gene of MYB89 (Li et al., 2017a).

## Regulatory Mechanisms Mediating WRI1 Protein Stability and Activity

WRI1 activity is vital for oil biosynthesis as well as some developmental processes, such as seed germination and seedling establishment (Focks and Benning, 1998; Cernac and Benning, 2004; Cernac et al., 2006). Hence, it is not surprising that recent work uncovered several regulatory mechanisms that modulate WRI1 activity at different levels.

To prevent hyper-activation of key biological processes, the activities of transcription factors controlling metabolic pathways are often fine-tuned through protein-interacting regulators (Mouchiroud et al., 2014; Pireyre and Burow, 2015; Hafner et al., 2019). The interaction of AtWRI1 with CULLIN3-based E3 ligase adaptor BTB/POZMATH 1 (BPM1) in yeast-two-hybrid (Y2H) screening identified BPM1 as an AtWRI1-interacting partner (Chen et al., 2013). It is also demonstrated that AtWRI1 physically interacts with other BPM proteins. AtWRI1 assembles with E3 ligase adaptor BPMs, which mediates the degradation of AtWRI1 *via* 26S proteasome (Chen et al., 2013), connecting E3 ligase with plant oil production through the mediation of WRI1 protein stability.

Many proteins lack fixed three-dimensional structures, and intrinsic disordered regions (IDRs) have been found in eukaryotic proteins (Dyson and Wright, 2005; Kragelund et al., 2012; Valsecchi et al., 2013). IDRs tend to facilitate different confirmation requirements for varied specificities that are often regulated by post-translational modifications. Recent *in silico* analysis predicted three IDRs in the AtWRI1 protein (Ma et al., 2015). Functional analysis led to the characterization of an IDR3-located PEST motif (related to proteolysis), and a transactivation domain (TAD). Both TAD and IDR3-PEST motif are in the C-terminal region of AtWRI1, although not overlapping (**Figure 1A**). AtWRI1 variants, including IDR3-PEST deletion and mutations in putative phosphorylation residues of the IDR3-PEST motif, display enhanced protein stability and the expression of these variants elevated oil biosynthesis compared to that of AtWRI1 WT (Ma et al., 2015); therefore, phosphorylation is speculated to regulate AtWRI1 stability and activity (Ma et al., 2015).

In recent years, new AtWRI1-interacting regulators have been identified, expanding our understanding of the AtWRI1 regulatory network (summarized in **Figure 1B**). AtWRI1 physically interacts with 14-3-3 proteins in yeast and plant cells (Ma et al., 2016). 14-3-3 proteins are phosphopeptide-binding factors that are involved in various biological and physiological processes, such as metabolism, membrane transport, signal transduction, and protein modification (Jaspert et al., 2011; Paul et al., 2012; de Boer et al., 2013). Overexpression of *14-3-3* elevated oil production by increasing transcriptional activity and protein stability of AtWRI1 (Ma et al., 2016). Subsequent functional analysis identified overlapping 14-3-3- and BPM- binding motifs in AtWRI1. A hypothetical model suggests the interaction between AtWRI1 and 14-3-3 hinders either BPMs binding to AtWRI1 or the detachment of BPMs from AtWRI1. The AtWRI1–14-3-3 complex possesses elevated stability and transcriptional activity (Ma et al., 2016; Kong and Ma, 2018a).

It is often the case that plant gene regulation does not require *de novo* synthesis of transcription factor proteins; rather, it is mediated by post-transcriptional modification pathways. Among such pathways, protein phosphorylation mediated by kinases is perhaps the best studied process. KIN10 is an SNF1-related protein kinase which serves as a central regulator in transcriptional control of plant energy signal transduction pathways (Baena-Gonzalez et al., 2007). Recently, KIN10 has been shown to physically interact with and phosphorylate AtWRI1, leading to AtWRI1 degradation (Zhai et al., 2017). Phosphorylation deficient mutations at two residues in AtWRI1 (T70 and S166) impair KIN10-mediated phosphorylation and increase the AtWRI1 stability. 14-3-3 binding motif and KIN10 phosphorylation sites in AtWRI1 are neighboring, suggesting the possibility that the two may have overlapping proteasome-mediated protein degradation pathways (Zhai et al., 2017). In addition, trehalose 6-phosphate (T6P) was found to contribute to WRI1 stabilization and increase the generation of fatty acids *via* suppression of KIN10 activity (Zhai et al., 2018).

Recruitment of mediator complexes by transcription factors to accomplish their functions is a conserved mechanism of transcriptional regulation in eukaryotes (Taatjes, 2010). Physical interaction of Arabidopsis mediator complex MED15 subunit with AtWRI1 was demonstrated *in vivo* and *in vitro* (Kim et al., 2016). Transgenic Arabidopsis plants overexpressing *MED15* displayed increased expression levels of oil biosynthetic genes targeted by AtWRI1. ChIP assays revealed that MED15 is capable of binding to promoters of AtWRI1 targets (Kim et al., 2016). However, the elevated expression of these AtWRI1 target genes in transgenic *wri1* plants overexpressing *MED15* suggested that MED15 interacts with other transcriptional regulators to regulate the gene expression of AtWRI1 targets (Kim et al., 2016).

## Functional Conservation of WRI1 in Different Plant Species

A high degree of cross-species conservation is a hallmark of important master transcriptional regulators. A number of *WRI1* orthologs have been found in various monocot and dicot plant species, including *Brassica napus* (Liu et al., 2010), *Brachypodium distachyon* (Yang et al., 2015), *Camelina sativa* (An et al., 2017), *Zea mays* (Shen et al., 2010; Pouvreau et al., 2011), *Elaeis guineensis* (Bourgis et al., 2011; Ma et al., 2013), *Glycine max* (Manan et al., 2017; Zhang et al., 2017; Chen et al., 2020), *Ricinus communis* (Tajima et al., 2013; Ji et al., 2018), *Avena sativa* (Grimberg et al., 2015), *Cocos nucifera* (Sun et al., 2017), *Cyperus esculentus* (Grimberg et al., 2015), *Gossypium spp* (Qu et al., 2012), *Jatropha curcas* (Ye et al., 2018), *Persea americana* (Kilaru et al., 2015), *Solanum tuberosum* (Grimberg et al., 2015), and *Oryza sativa* (Mano et al., 2019). Expressing *AtWRI1* or *WRI1* orthologs in *wri1* loss-of-function mutants complemented the decreased seed oil phenotype of *wri1* (Cernac and Benning, 2004; Pouvreau et al., 2011; Ma et al., 2013; An et al., 2017; Ye et al., 2018; Chen et al., 2020). Numerous *WRI1* orthologs display high expression levels in developing seeds, correlating closely to the *AtWRI1* expression patterns. Nonetheless, some *WRI1s* have been found to show high expression in non-seed tissues. For example, the expression of oil palm *WRI1* (*EgWRI1*) is high in mesocarp and substantially increases in the ripening processes of fruits (Bourgis et al., 2011). In addition, *PaWRI1* and *CeWRI1* exhibit high expression levels in avocado mesocarp and nutsedge stem tubers, respectively (Grimberg et al., 2015; Kilaru et al., 2015). Structural features and functional domains/motifs, which have been identified in the AtWRI1 protein, including “VYL” (Ma et al., 2013), IDR (Ma et al., 2015), and the PEST motif (Ma et al., 2015), are conserved in other WRI1 proteins (Ma et al., 2013; Ma et al., 2015; Yang et al., 2015; An et al., 2017; Kong and Ma, 2018b; Ye et al., 2018; Chen et al., 2020; Kong et al., 2019; Snell et al., 2019; Tang et al., 2019). *WRI1* orthologs identified in *Ricinus connunis and Oryza sativa* encode WRI1 proteins lacking a “VYL” motif but still trigger oil biosynthesis *in planta* by activating the expression of WRI1 target genes (Ji et al., 2018; Mano et al., 2019). Recent work on RcWRI1-B and OsWRI1-1 suggested that “VYL” might not be important for some WRI1s (Ji et al., 2018; Mano et al., 2019), despite its conservation in numerous WRI1s and its functional importance in both AtWRI1 (Ma et al., 2013) and Arabidopsis AP2 transcription factor, ANT (Krizek, 2003).

## WRI1 Target Genes Which are Not Involved in Oil Biosynthesis

In addition to its role as a transcriptional regulator controlling expression of genes in late glycolysis and oil biosynthesis, WRI1 has been found to target genes in pathways other than oil biosynthesis. AtWRI1 is not only able to bind to the promoters of *PINs* (*PIN-FORMEDs*) through the AW-box, but also binds to the promoter of *GH3.3* (a gene involved in auxin degradation) *via* a non-AW-box element, suggesting a role in modifying auxin homeostasis (Kong et al., 2017) (also see **Figure 1B**). The altered expression of auxin-related genes was also found in roots of soybean *GmWRI1s*-overexpressing plants or *GmWRI1s*-RNAi plants compared to WT control (Chen et al., 2020). Furthermore, *AtWRI1* is a homolog of *CitAP2.10* (encoding a *Citrus sinensis* AP2 transcription factor which is in charge of (+)-valencene biosynthesis), and AtWRI1 is capable of transactivating the *C. sinensis terpene synthase* 1 (*CsTPS1*) promoter in a dual-luciferase assay (Shen et al., 2016). How regulation of the AtWRI1 targets is linked to plant growth and development, such as root growth (Kong et al., 2017), is currently under investigation. It is important to consider the alternative targets when utilizing WRI1 to bioengineer crops for plant oil production. However, convincing evidence exists demonstrating *WRI1* as one of the most effective genes for plant oil bioengineering.

## Importance of WRI1 for Plant Oil Production

Transgenic plants overexpressing *AtWRI1* or *WRI1* orthologs show significantly increased seed oil content in a number of studies (Cernac and Benning, 2004; Liu et al., 2010; Shen et al., 2010; An and Suh, 2015; Yang et al., 2015; Sun et al., 2017; Ye et al., 2018). Transgenic Arabidopsis and Brachypodium seedlings, overexpressing *AtWRI1* and *BdWRI1* respectively, have increased oil content in vegetative tissues (Sanjaya et al., 2011; Yang et al., 2015). Selection of proper promoters to drive *WRI1* expression is also critical for oil bioengineering. For instance, significantly increased oil content in seeds was observed in transgenic maize with embryo-preferred *OLEOSIN* (*OLE*) promoter driven *ZmWRI1*, but there was no distinguishable oil increase in transgenic maize with the starch endosperm-specific *19 KD ZEIN* promoter driven *ZmWRI1* (Shen et al., 2010). Another example is transgenic Arabidopsis transformed by a vector that confers *FUS3* promoter driven *AtWRI1* expression, which turns out to be effective in increasing seed oil production due to an extension of oil biosynthesis during the mid-phase seed developmental process (Kanai et al., 2016).

Ectopic expression of *AtWRI1* or *WRI1* orthologs in a transient tobacco expression system is also able to productively trigger TAG accumulation in tobacco leaves (Vanhercke et al., 2013; Grimberg et al., 2015; Ma et al., 2015). Transient co-expression of *AtWRI1* and *DGAT1* in tobacco leaves has led to considerably augmented oil content compared to expression of *WRI1* alone, which suggests a synergistic effect between these two genes (Vanhercke et al., 2013). Transient overexpression of engineered *AtWRI1* variants in tobacco leaves, either deletion of IDR3-PEST motif or phosphorylation deficient mutations in the IDR3-PEST motif, resulted in production of stabilized WRI1 proteins and enhanced oil production (Ma et al., 2015). Transient co-expression of *AtWRI1* and *14-3-3* in tobacco leaves resulted in increased stability of AtWRI1 and enhanced oil production (Ma et al., 2016). The AtWRI1^K2RK3R^ variant, with mutations in two ubiquitination residues, resulted in increased protein stability and oil biosynthesis using a tobacco transient expression system (Zhai et al., 2017). In metabolic engineering to enhance oil production in vegetative tissues, the strategy of “push, pull, package, and protect” is to upregulate (push) the *de novo* fatty acid pathway using a key transcription factor, combined with pulling the precursors toward the end products using rate-limiting enzymes, packaging TAG in oil bodies, and protecting TAG from degradation. *WRI1* overexpression has been shown to be pivotal in such designed strategy (Vanhercke et al., 2019).

Transgenic plants, overexpressing transcription factors which can upregulate *WRI1* expression, also display elevated seed oil content. For instance, transgenic Arabidopsis plants that overexpress *GmZF351* or *GmDREBL* have increased oil content in seeds. Both soybean transcription factors are capable of binding to the *AtWRI1* promoter and activate *AtWRI1* expression (Zhang et al., 2016b; Li et al., 2017b). Overexpression of *ZmLEC1* (an activator of *WRI1*) also effectively increases seed oil in various plants species including Arabidopsis, Camelina, and maize (Shen et al., 2010; Zhu et al., 2018).

Unusual fatty acids, such hydroxy fatty acids (HFAs), have high value for industrial applications because of their distinctive physical and chemical properties. Nonetheless, transgenic Arabidopsis plants which overexpress a hydroxylase gene only yield low amount of HFA with reduction of total seed oil (Bates and Browse, 2011; Bates et al., 2014). A feedback inhibition of fatty acid biosynthesis was believed to be the main reason for the decreased seed oil content (Bates et al., 2014). In order to overcome this bottleneck, Adhikari et al. attempted to generate Arabidopsis transgenic plants which co-express *AtWRI1* with *RcFAH12* (encoding a castor fatty acid hydroxylase). The resulting transgenic Arabidopsis plants have significantly elevated proportions of HFA and entire seed oil content, demonstrating WRI1’s role for effective circumventing feedback inhibition of fatty acid synthesis when ectopic expressing a hydroxylase gene (Adhikari et al., 2016).

Aberrant growth or cell death was observed in some transgenic plants which overexpress *WRI1* (Cernac and Benning, 2004; Marchive et al., 2014; Yang et al., 2015). Choosing suitable promoters for certain plant species hence becomes a necessary consideration for the development of oil crops.

## Perspectives

Since the discovery of the *wri1-1* mutant, multiple studies have established the central role of WRI1 in transcriptional regulation of plant oil biosynthesis. However, key questions remain, including what are the upstream regulators of WRI1 and how do the master regulators of seed development, such as LEC1, LEC2, and FUS3, mediate the expression of *WRI1*? Alternative transcriptional regulators could play important roles in controlling *WRI1* expression in response to various developmental or environmental cues. Several transcriptional regulators of *EgWRI1* have been recently identified in oil palm (Yeap et al., 2017). Whether the newly expanded regulatory network in oil palm is conserved in other plant species is unclear. More detailed analyses of the *WRI1* promoters from various plant species may allow us to generalize the conserved nature of combinatorial WRI1 transcriptional regulation.

AtWRI3 and AtWRI4 are WRI1-like proteins identified in Arabidopsis. Seed-specific overexpression of *AtWRI3* or *AtWRI4* in the *wri1* mutant complemented the low seed-oil phenotype (To et al., 2012). AtWRI3 and AtWRI4 seem to share overlapping function with AtWRI1 in providing acyl precursors for cutin biosynthesis in floral tissues; however, their roles in seed oil biosynthesis are less certain as they are predominantly expressed in non-seed tissues such as stems and flowers (To et al., 2012). It should be noted that the *wri1 wri3 wri4* triple mutant displayed no apparent changes in vegetative growth, raising the speculation of the involvement of other unidentified transcriptional regulators that control fatty acid biosynthesis in vegetative tissues (To et al., 2012; Marchive et al., 2014). Investigation of the unique and overlapping functions of WRI1-like factors will shed light on the complex transcriptional control of fatty acid biosynthesis, especially in vegetative tissues.

Structural analyses of WRI1 variants and orthologs have revealed that the IDR3-PEST motif, binding motifs of E3 ligase adaptor BPMs and 14-3-3s, and phosphorylation residues (T70/S166) are relevant for AtWRI1 stability and transcriptional activity, consequently affecting plant oil production (Ma et al., 2013; Ma et al., 2015; Ma et al., 2016; Zhai et al., 2017). Recent studies on the missing “VYL” motif of RcWRI1-B and OsWRI1-1 also open the discussion about the importance of “VYL” in WRI1 proteins (Ji et al., 2018; Mano et al., 2019). Generation of the three-dimensional structure, in combination of computer modeling, will further advance our understanding of WRI1 function.

The emerging picture shows crosstalk between the WRI1-interacting regulators which are also post-translationally regulated, highlighting the cooperative activity of the WRI1 transcriptional machinery (**Figure 1***)*. Phosphorylation has been proposed to play dual roles in mediating AtWRI1 protein stability during embryo development (Ma et al., 2016; Kong and Ma, 2018a). Therefore, one possible mechanism to fine-tune WRI1 activity is through phosphorylation and dephosphorylation by various kinases and phosphatases in response to different developmental and environmental cues. Recent progress in WRI1 interactome facilitates the identification of novel WRI-interacting factors; however, the molecular mechanisms mediating the interplay among these regulators remain to be elucidated.

Key questions also remain regarding WRI1 protein stability. Why do plants degrade WRI1? Understanding of the control mechanism of WRI1 degradation, particularly under different developmental stages and environmental conditions, will help address the biological questions of “when” and “how” the endogenous degradation system activates to degrade WRI1. The answers to these questions may ultimately be used to guide our efforts to increase plant oil production.

AtWRI1 activates the expression of several *BIOTIN ATTACHMENT DOMAIN-CONTAINING* (*BADC*) genes that inhibit fatty acid biosynthesis (Liu et al., 2019), suggesting an additional layer of regulation of WRI1-mediated fatty acid biosynthesis. In summary, while the recent insights on WRI1 significantly advance our understanding of the molecular mechanisms governing plant oil biosynthesis, our knowledge of the complex regulatory system is far from complete.

## Author Contributions

QK, YY, and WM conceived the ideas, prepared the figures and wrote the first draft. LG, LY, and WM reviewed and edited the figures and manuscript. All authors read and approved the final version of the manuscript.

## Funding

This work was supported by a Nanyang Technological University Startup grant and a Ministry of Education (MOE) of Singapore Tier 1 to WM (2018-T1-002-019).

## Conflict of Interest

The authors declare that the research was conducted in the absence of any commercial or financial relationships that could be construed as a potential conflict of interest.
